# Surveillance of respiratory viruses at health facilities from across Kenya, 2014

**DOI:** 10.12688/wellcomeopenres.17908.2

**Published:** 2022-09-28

**Authors:** Nickson Murunga, Bryan Nyawanda, Joyce U. Nyiro, Grieven P. Otieno, Everlyn Kamau, Charles N. Agoti, Clement Lewa, Alex Gichuki, Martin Mutunga, Nancy Otieno, Lilian Mayieka, Melvin Ochieng, Gilbert Kikwai, Elizabeth Hunsperger, Clayton Onyango, Gideon Emukule, Godfrey Bigogo, Jennifer R. Verani, Sandra S. Chaves, D. James Nokes, Patrick K. Munywoki

**Affiliations:** 1 KEMRI-Wellcome Trust Research Programme, Kilifi, Kenya; 2 KEMRI-Centre for Global Health Research, Kisumu, Kenya; 3 U.S. Centers of Disease Control and Prevention (CDC), Center for Global Health, Division of Global Health Protection, Nairobi, Kenya; 4 School of Life Sciences and Zeeman Institute for Systems Biology and Infectious Disease Epidemiology Research (SBIDER), University of Warwick, Coventry, CV4 7AL, UK

**Keywords:** human rhinoviruses, respiratory syncytial viruses, influenza viruses, endemic human coronaviruses, adenoviruses, multiplex RT-PCR, nasopharyngeal swab, oropharyngeal swab

## Abstract

**Background:** Acute respiratory illnesses (ARI) are a major cause of morbidity and mortality globally. With (re)emergence of novel viruses and increased access to childhood bacterial vaccines, viruses have assumed greater importance in the aetiology of ARI. There are now promising candidate vaccines against some of the most common endemic respiratory viruses. Optimal delivery strategies for these vaccines, and the need for interventions against other respiratory viruses, requires geographically diverse data capturing temporal variations in virus circulation.

**Methods:** We leveraged three health facility-based respiratory illness surveillance platforms operating in 11 sites across Kenya. Nasopharyngeal (NP) and/or oropharyngeal (OP) specimens, patient demographic, and clinical characteristics were collected in 2014 from individuals of various ages presenting with respiratory symptoms at the surveillance facilities. Real time multiplex polymerase chain reaction was used to detect rhinoviruses, respiratory syncytial virus (RSV), influenza virus, human coronaviruses (hCoV), and adenoviruses.

**Results:** From 11 sites, 5451 NP/OP specimens were collected and tested from patients. Of these, 40.2% were positive for at least one of the targeted respiratory viruses. The most frequently detected were rhinoviruses (17.0%) and RSV A/B (10.5%), followed by influenza A (6.2%), adenovirus (6.0%) and hCoV (4.2%). RSV was most prevalent among infants aged <12 months old (18.9%), adenovirus among children aged 12–23 months old (11.0%), influenza A among children aged 24–59 months (9.3%), and rhinovirus across all age groups (range, 12.7–19.0%). The overall percent virus positivity varied by surveillance site, health facility type and case definition used in surveillance.

**Conclusions:** We identify rhinoviruses, RSV, and influenza A as the most prevalent respiratory viruses. Higher RSV positivity in inpatient settings compared to outpatient clinics strengthen the case for RSV vaccination. To inform the design and delivery of public health interventions, long-term surveillance is required to establish regional heterogeneities in respiratory virus circulation and seasonality.

## Introduction

Globally, acute respiratory illnesses, including pneumonia, are the leading cause of morbidity and mortality especially among children younger than five years ^1^. In 2019 alone, 14% of all deaths in children less than five years were attributed to pneumonia, with countries with less access to healthcare resources bearing the greatest burden ^2^. With the recent introduction and improved access to childhood bacterial vaccines such as *Haemophilus influenzae* type b, pertussis and pneumococcal conjugate vaccines (PCV), the role of respiratory virus interventions has assumed a greater importance ^3– 6^. A multi-country study by the Pneumonia Etiology Research for Child Health (PERCH) group in sub-Saharan Africa and South Asia reported that 61% of hospitalized pneumonia cases were due to viral pathogens with respiratory syncytial virus (RSV) having the greatest aetiological role at 31% of all pathogens ^6^. Influenza viruses and human coronaviruses were also identified as important causes of severe pneumonia ^6^.

Though licensed vaccines exist against seasonal influenza viruses ^7^, most low- and middle-income countries (LMICs) have not established an influenza vaccination program ^8^. RSV now has promising vaccine products that are coming up soon for licensure ^9, 10^. Furthermore, the pharmaceutical industry, Gavi and World Health Organisation (WHO) have been considering vaccines for other respiratory viruses, especially following the successful development and launch of the COVID-19 vaccines using new platforms (vector-based and mRNA vaccines) ^11^. The prediction of the impact and how best to use the existing and new vaccines requires a detailed understanding of how the viruses spread, and how the patterns of disease change over time in various regions and age groups ^12^. 

In this paper, we describe the detection of several respiratory viruses over one calendar year in multiple health facility-based surveillance sites across Kenya. Description of the patterns of virus circulation across the country may assist local and international public health authorities to target prevention measures more effectively against existing and emerging respiratory pathogens.

## Methods

### Surveillance sites

**Figure 1.**
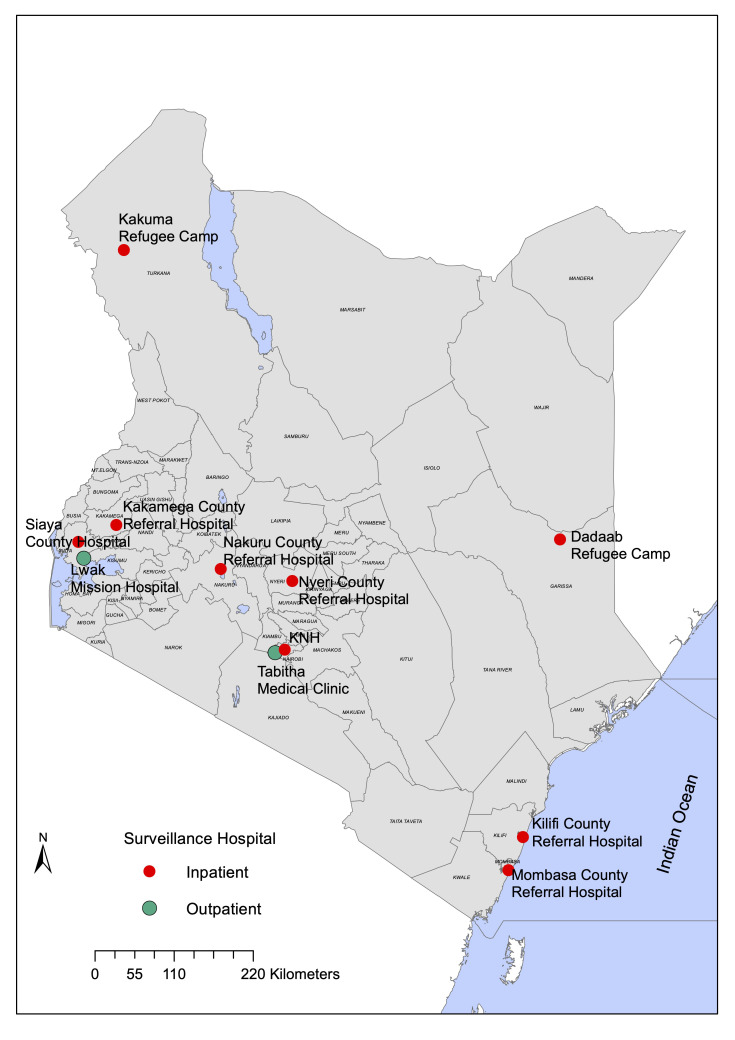
Kenyan map showing the location of the 11 surveillance sites.

Data originated from 11 study sites across Kenya ( Figure 1 and Table 1) under three platforms which include eight influenza sentinel surveillance hospitals ^13– 15^, and two outpatient clinics in Asembo and Kibera under Population-Based Infectious Disease Surveillance (PBIDS) ^16^ and a paediatric surveillance at Kilifi County Hospital (KCH) ^17^. The influenza sentinel surveillance was established by Kenya Medical Research Institute-Centre for Global Research (KEMRI-CGHR), US Centers for Disease Control and Prevention (CDC)-Kenya and Ministry of Health as part the of Global Influenza Program and have been in operation since 2006 ^13^. The PBIDS platform in Asembo and Kibera is run by KEMRI-CGHR with financial and technical support from CDC since 2006 ^16^. Surveillance at KCH is conducted by KEMRI-Centre for Geographic Medical Research Coast (CGMRC) under KEMRI-Wellcome Trust Research Programme (KWTRP) in Kilifi, Kenya ^17^. Overall, the selected surveillance hospitals offer a good representation of the various ecological settings including urban and rural communities in Kenya.

**Table 1.** Description of the 11 respiratory surveillance sites across Kenya.

	Surveillance site	Region	Setting	Inclusion criteria	Age included
1	Tabitha Medical Clinic, Kibera PBIDS	Central	Outpatient	ALRTI ^3^ & ILI ^2^	All
2	Kenyatta National Hospital	Central	Inpatient	SARI ^1^ & ILI ^2^	<13 years
3	Nyeri County Referral Hospital	Central	Inpatient	SARI ^1^	All
4	Mombasa County Referral Hospital	Coast	Inpatient	SARI ^1^	All
5	Kilifi County Referral Hospital	Coast	Inpatient	Pneumonia ^4^	<5 years
6	Kakuma Refugee Camp	North	Inpatient	SARI ^1^	All
7	Dadaab Refugee Camp	North	Inpatient	SARI ^1^	All
8	Nakuru County Referral Hospital	Rift valley	Inpatient	SARI ^1^	All
9	Kakamega County Referral Hospital	Western	Inpatient	SARI ^1^	All
10	Lwak Mission Hospital, Asembo PBIDS*	Western	Outpatient ^3^	ALRTI ^3^ & ILI ^2^	All
11	Siaya County Hospital	Western	Inpatient	SARI ^1^	All

Key: 1, SARI, Severe Acute Respiratory Illness is defined as an acute respiratory illness requiring hospitalization with a history of fever or measured fever ≥38°C **AND** a cough with an onset of symptoms within the last 14 days; 2, ILI, Influenza Like Illness is defined as measured fever of ≥38°C OR sore throat in an outpatient of any age, ALRTI, Acute lower respiratory tract illness is defined as presence of cough **OR** difficulty in breathing with one of the following danger signs: chest in-drawing, stridor, unable to breastfeed, vomit everything, convulsions, lethargy, or unconsciousness ^13^; 4, Pneumonia is defined as modified WHO syndromic severe or very severe pneumonia ^17^; PBIDS, Population-Based Infectious Disease Surveillance.

### Patient enrollment

The surveillance sites recruited patients of various ages presenting with clinical features of an acute respiratory illness with a measured fever of ≥38°C AND a cough with an onset of symptoms within the last seven days (influenza-like illness, ILI) or acute respiratory illness requiring hospitalization with a history of fever or measured fever ≥38°C **AND** a cough with an onset of symptoms within the last 14 days (severe acute respiratory illness, SARI) or acute lower respiratory tract illness (ALRTI) defined as presence of cough OR difficulty in breathing with one of the following danger signs: chest in-drawing, stridor, unable to breastfeed, vomit everything, convulsions, lethargy, or unconsciousness, or an adaptation of WHO severe/very severe pneumonia as defined in Table 1. Despite the different case definitions, they were consistently applied within each platform over the course of the study period, 1 ^st^ January to 31 ^st^ December 2014. The funding limitations restricted the study period to one year.

### Data and specimen collection

Patient demographic data as well as clinical features of the presenting illness were collected from the 11 sentinel surveillance sites in real time to custom designed databases using tablets or desktop computers. Additional data were collected on discharge to record illness outcome. Nasopharyngeal (NP) and/or oropharyngeal (OP) swabs were collected from eligible patients and stored in viral transport media. Upon collection all specimens were immediately stored in a cool box (with ice packs) before being transported to the laboratory for long-term storage at -70°C freezer. The specimens were retrieved and tested for additional respiratory viruses in 2016 at the Virus Epidemiology and Control Research Group laboratory at KWTRP in Kilifi, Kenya.

### Specimen testing

Using previously described methods ^18– 20^, ribonucleic acid (RNA) was extracted from the respiratory specimens by Qiacube HT using RNeasy extraction kit (Qiagen, Germany, catalogue number 74171) from 140μl of the swab sample with a 10-minute incubation offboard lysis according to the manufacturer’s instruction and screened for RSV (groups A and B), rhinovirus (RV), human coronaviruses (hCoV-OC43, -NL63, -229E), influenza virus (Influenza virus type A, Influ-A), and adenovirus (ADV). A multiplex 7500 real time PCR assay system from Applied Biosystems based on QuantiFast Multiplex RT-PCR Kit with ROX (catalogue number 204954) was used. The dye/fluorescence marker used for detection was VIC™, FAM™, and Cy5™. Cycling parameters used were 50°C for 20 minutes, 95°C for 5 minutes, 40 cycles of 95°C for 15 seconds and 40 cycles of 60°C for 30 seconds. Influenza testing for specimens from the eight influenza surveillance sites was performed at the CDC-supported laboratories at KEMRI-CGHR, Kisumu and Nairobi. Samples with cycle threshold (Ct) of <35.0 were defined as positive for the target virus.

### Statistical analysis

Statistical analysis was conducted using STATA version 15.1 (College Station, Texas) (RRID:SCR_012763). The clinic and laboratory data were merged with the demographic data with the final analytical dataset consisting of participants with respiratory symptoms and with fully linked data. Summary statistics on percent virus positives by age and site were computed. Chi-squared and Mann-Whitney tests were used to test associations of virus occurrence with age, calendar month, facility, setting (outpatient or inpatient) and other patient characteristics. Frequency distribution graphs were generated for all virus targets. Overall and site-specific monthly prevalence for each virus were generated and compared. Sites from the same geographical areas were grouped into regions as shown in Table 1 to explore regional variations.

## Ethical considerations

All individuals, parents and guardians gave written informed consent for themselves or their children to participate in the original studies. The study was approved by the KEMRI-Scientific and Ethical Review Unit (SSC #3044) and CDC Institutional Review Board (#6806) to use pre-existent, pseudonymized specimens and data. All the studies had ethical approval for specimens to be tested for a broad range of respiratory pathogens.

## Results

### Baseline characteristics

**Figure 2.**
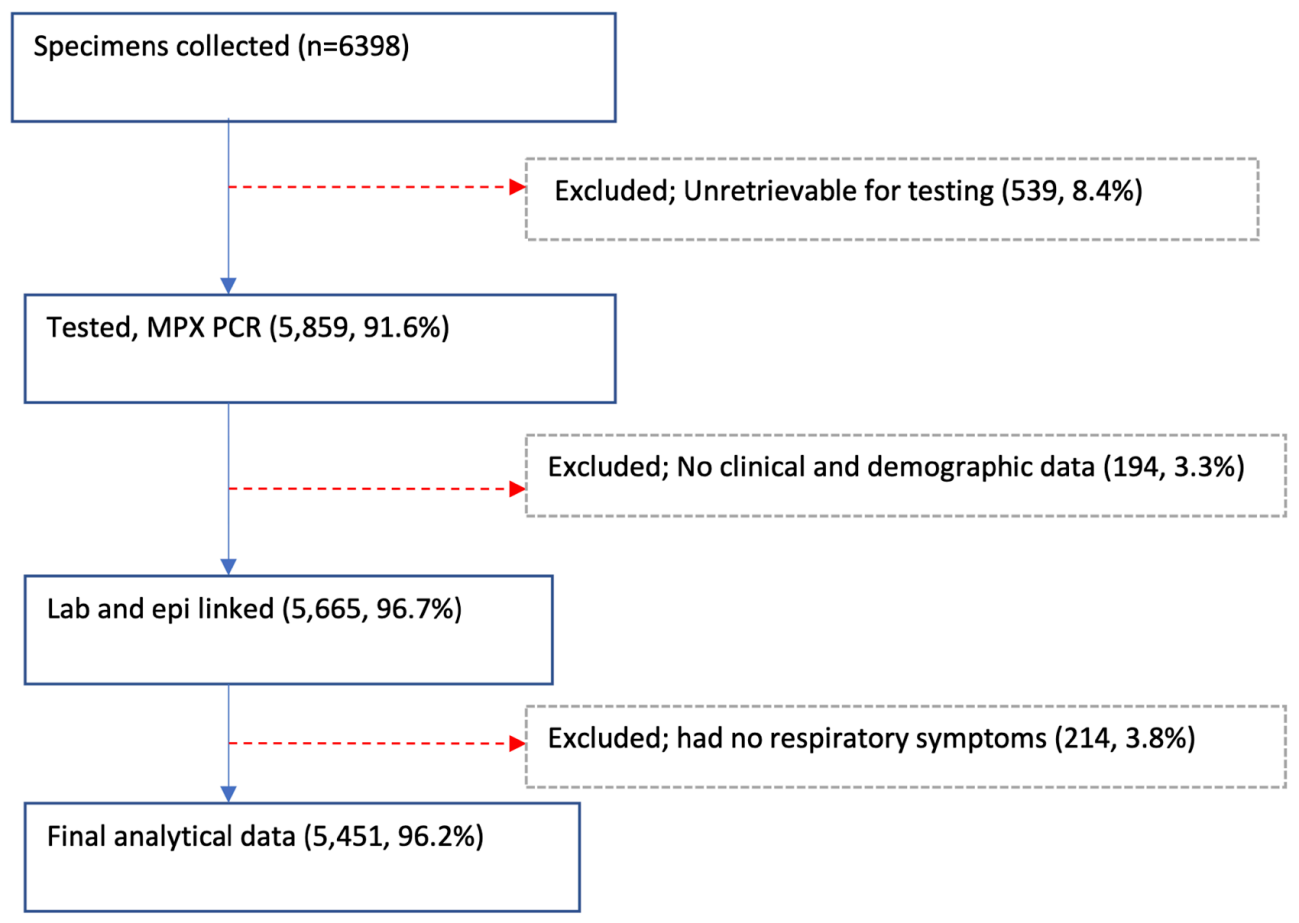
Flow diagram showing the specimen collections, testing, and linkage with epidemiologic (epi) data from the 11 surveillance sites in Kenya, 2014.

A total of 6398 NP and/or OP swabs were collected from patients with acute respiratory illnesses at the 11 sites in 2014 with 5859 (91.6%) available and tested, Table 2 and Figure 2. Of the tested specimens, 5665 (96.7%) were linked with their respective demographic and clinical data. Excluding 214 samples collected from Siaya and Kibera patients with missing data on respiratory symptoms, the final analytical dataset comprised of 5451 specimens, of which 2863 (52.5%) were from male participants. The average number of samples per site was 496, ranging from 154 in Dadaab to 862 in Asembo ( Table 3).

**Table 2.** Nasopharyngeal and oropharyngeal (NP/OP) specimen collections, testing and linkage with clinic and demographic data in the 11 surveillance sites in Kenya, 2014.

Sites	N	Tested		Linkage complete		With respiratory symptoms ^1^	
		n	%	n	%	n	%
Dadaab	189	158	83.6	154	97.5	154	100.0
KNH	510	357	70.0	343	96.1	343	100.0
Kakamega	464	418	90.1	402	96.2	402	100.0
Kakuma	220	175	79.5	175	100.0	175	100.0
Kibera	640	631	98.6	631	100.0	569	90.2
Kilifi	722	722	100.0	722	100.0	722	100.0
Asembo	879	862	98.1	862	100.0	862	100.0
Mombasa	524	455	86.8	445	97.8	445	100.0
Nakuru	811	714	88.0	712	99.7	712	100.0
Nyeri	482	427	88.6	425	99.5	425	100.0
Siaya	936	922	98.5	794	86.1	642	80.9
Not recorded	21	18	85.7	0	0.0	0.0	0.0
**Total**	**6398**	**5859**	**91.6**	**5665**	**96.7**	**5451**	**96.2**

1, Excluded cases with NP/OP collections from patients without documented respiratory symptoms.

**Table 3.** Baseline characteristics of patients with respiratory symptoms by the surveillance site in Kenya, 2014.

Characteristic	Dadaab (n=154)		KNH (n=343)		Kakamega (n=402)		Kakuma (n=175)		Kibera (n=569)		Kilifi (n=722)		Asembo (n=862)		Mombasa (n=445)		Nakuru (n=712)		Nyeri (n=425)		Siaya (n=642)		Total (n=5451)	
	n	%	n	%	n	%	n	%	n	%	n	%	n	%	n	%	n	%	n	%	n	%	n	%
Gender																								
Female	60	39.0	150	43.7	186	46.3	76	43.4	284	49.9	314	43.5	449	52.1	185	41.6	342	48.0	170	40.0	372	57.9	2588	47.5
Male	94	61.0	193	56.3	216	53.7	99	56.6	285	50.1	408	56.5	413	47.9	260	58.4	370	52.0	255	60.0	270	42.1	2863	52.5
Age																								
<12m	61	39.6	205	59.8	93	23.1	50	28.6	54	9.5	475	65.8	92	10.8	226	50.8	287	40.3	143	33.7	95	14.8	1781	32.7
12– 23m	39	25.3	84	24.5	71	17.7	85	48.6	82	14.4	107	14.8	136	15.8	129	29.0	137	19.2	107	25.2	69	10.8	1046	19.2
24– 59m	26	16.9	52	15.2	131	32.6	32	18.3	163	28.7	122	16.9	264	30.6	76	17.1	191	26.8	144	33.9	95	14.8	1296	23.8
5 – 14y	20	13.0	2	0.6	74	18.4	8	4.6	180	31.6	18	2.5	219	25.4	13	2.9	35	4.9	15	3.5	47	7.3	631	11.6
15 – 44y	6	3.9	0	0	18	4.5	0	0	80	14.1	0	0	107	12.4	1	0.2	54	7.6	9	2.1	233	36.3	508	9.3
45 + y	2	1.3	0	0	15	3.7	0	0	10	1.8	0	0	44	5.1	0	0	8	1.1	7	1.7	103	16.0	189	3.5
Symptoms																								
Cough	153	99.4	338	98.5	402	100	174	99.4	478	84.0	571	79.1	824	95.6	445	100	712	100	425	100	560	87.2	5082	93.2
Difficulty in breathing	-	-	228	66.5	127	31.6	-	-	7	1.2	-	-	45	5.2	298	67.0	436	61.2	45	10.6	369	57.5	1555	28.5
Runny nose	-	-	16	4.7	256	63.7	-	-	230	40.4	-	-	496	57.5	322	73.4	322	72.4	173	40.7	-	-	1815	33.3
Chest wall indrawing	-	-	207	60.4	56	13.9	-	-	14	2.5	577	79.9	11	1.3	196	44.0	197	27.7	19	4.5	-	-	1277	23.4
Wheeze	-	-	24	7.0	16	4.0	-	-	11	1.9	70	9.7	6	0.7	118	26.5	52	7.3	18	4.2	21	3.3	336	6.2
Lethargic	132	85.7	75	21.9	12	3.0	8	4.6	10	1.8	-	-	12	1.4	193	43.4	57	8.0	11	2.6	-	-	510	9.4

### Respiratory virus detections

Overall, 2193 (40.2%) of the tested specimens were positive for at least one of the target respiratory viruses. The median age of the virus-positive patients was 1.4 years (Interquartile range, IQR, 7 months – 3.3 years) and that of the virus-negative patients was 2.2 years (IQR, 10 months – 6.4 years) and the difference was statistically significant (p <0.001), Table 4. Infants (<1 year old) had the highest virus positivity at 38.5% while adults aged ≥ 45 years had the lowest at 1.4%. The percentage of specimens that were virus positive varied significantly by surveillance site and month of sampling. Inpatient facilities had higher virus-positivity relative to outpatient facilities, 43.9% vs. 30.0%, p-value <0.001), ( *Extended data*: Supplementary File, Supplementary Figure 1) ^21^. Specimens from individuals presenting with WHO syndromic severe or very severe pneumonia, SARI, SARI or ILI, and ILI had corresponding virus-positivity of 46.5%, 43.8%, 38.8%, and 30.0%, ( *Extended data*: Supplementary File, Supplementary Figure 2) ^21^.

**Table 4.** Baseline characteristics and virus detection among patients with respiratory symptoms from the 11 surveillance sites in Kenya, 2014.

Characteristic	Categories	Overall (N=5451)	Virus detection		P value ^2^
			Any positive (N=2193)	Negative (N=3258)	
Age in years	Median (IQR ^1^)	1.8 (0.75–4.8)	1.4 (0.63–3.3)	2.2 (0.84–6.4)	<0.001 ^3^
Age groups	<12m	1781 (32.7)	845 (38.5)	936 (28.7)	<0.001
	12– 23m	1046 (19.2)	461 (21.0)	585 (18.0)	
	24– 59m	1296 (23.8)	540 (24.6)	756 (23.2)	
	5 – 14y	631 (11.6)	164 (7.5)	467 (14.3)	
	15 – 44y	508 (9.3)	153 (7.0)	355 (10.9)	
	45 + y	189 (3.5)	30 (1.4)	159 (4.9)	
Male gender	n (%)	2863 (52.5)	1175 (53.6)	1688 (51.8)	0.2
Sites	Dadaab	154 (2.8)	55 (2.5)	99 (3.0)	<0.001
	KNH	343 (6.3)	133 (6.1)	210 (6.5)	
	Kakamega	402 (7.4)	148 (6.8)	254 (7.8)	
	Kakuma	175 (3.2)	74 (3.4)	101 (3.1)	
	Kibera	569 (10.4)	130 (5.9)	439 (13.5)	
	Kilifi	722 (13.3)	336 (15.3)	386 (11.9)	
	Asembo	862 (15.8)	300 (13.7)	562 (17.3)	
	Mombasa	445 (8.2)	214 (9.8)	231 (7.1)	
	Nakuru	712 (13.1)	353 (16.1)	359 (11.0)	
	Nyeri	425 (7.8)	216 (9.9)	209 (6.4)	
	Siaya	642 (11.8)	234 (10.7)	408 (12.5)	
Months	Jan	508 (9.3)	182 (8.3)	326 (10.0)	<0.001
	Feb	555(10.2)	241 (11.0)	314(9.6)	
	Mar	621(11.4)	220 (10.0)	401 (12.3)	
	Apr	429 (7.9)	184 (8.4)	245 (7.5)	
	May	493 (9.0)	198 (9.0)	295 (9.1)	
	Jun	492 (9.0)	232(10.6)	260 (8.0)	
	Jul	508 (9.3)	250 (11.4)	258 (7.9)	
	Aug	417 (7.7)	164 (7.5)	253 (7.8)	
	Sep	260 (4.8)	89 (4.1)	171 (5.3)	
	Oct	442 (8.1)	136 (6.2)	306 (9.4)	
	Nov	388 (7.1)	152 (6.9)	236 (7.2)	
	Dec	338 (6.2)	145 (6.6)	193 (5.9)	
Regions	Central	1337 (24.5)	479 (21.8)	858 (26.3)	<0.001
	Coast	1167 (21.4)	550 (25.1)	617 (18.9)	
	North	329 (6.0)	129 (5.9)	200 (6.14)	
	Rift Valley	712 (13.1)	353 (16.1)	359 (11.0)	
	Western	1906 (35.0)	682 (31.1)	1224 (37.6)	
Hospital Type	Inpatient	4020 (73.8)	1763 (80.4)	2257 (69.3)	<0.001
	Outpatient	1431 (26.3)	430 (19.6)	1001 (30.7)	

**Key: 1, IQR,** Interquartile range; 2, test statistic is a chi-square; 3, Mann-Whitney test used.

Of the 2193 virus detections, 1981 (90.3%) were single detections, 201 (9.2%) were dual, and 11 (0.5%) were triple. Rhinoviruses (147, 69.3%), adenoviruses (99, 46.7%), RSV A/B (75, 35.4%), hCoVs (65, 30.7%) and influenza viruses A (55, 25.9%) were co-detected in specimens with multiple viruses, ( *Extended data*: Supplementary File, Supplementary Figure 3) ^21^. The dual detected viruses were 52 (adenoviruses and rhinoviruses), 41 (RSV A/B and rhinoviruses), 25 (rhinoviruses and hCoV), 20 (rhinoviruses and influenza viruses A), 15 (adenoviruses and hCoV), 13 (RSV A/B and adenoviruses), 9 (adenoviruses and influenza viruses A), 9 (RSV A/B and influenza viruses A), 9 (RSV A/B and hCoV), and 8 (hCoV and influenza viruses A). For the triple detected viruses, the highest frequency was from six specimens with adenoviruses, hCoV and rhinoviruses.

**Figure 3.**
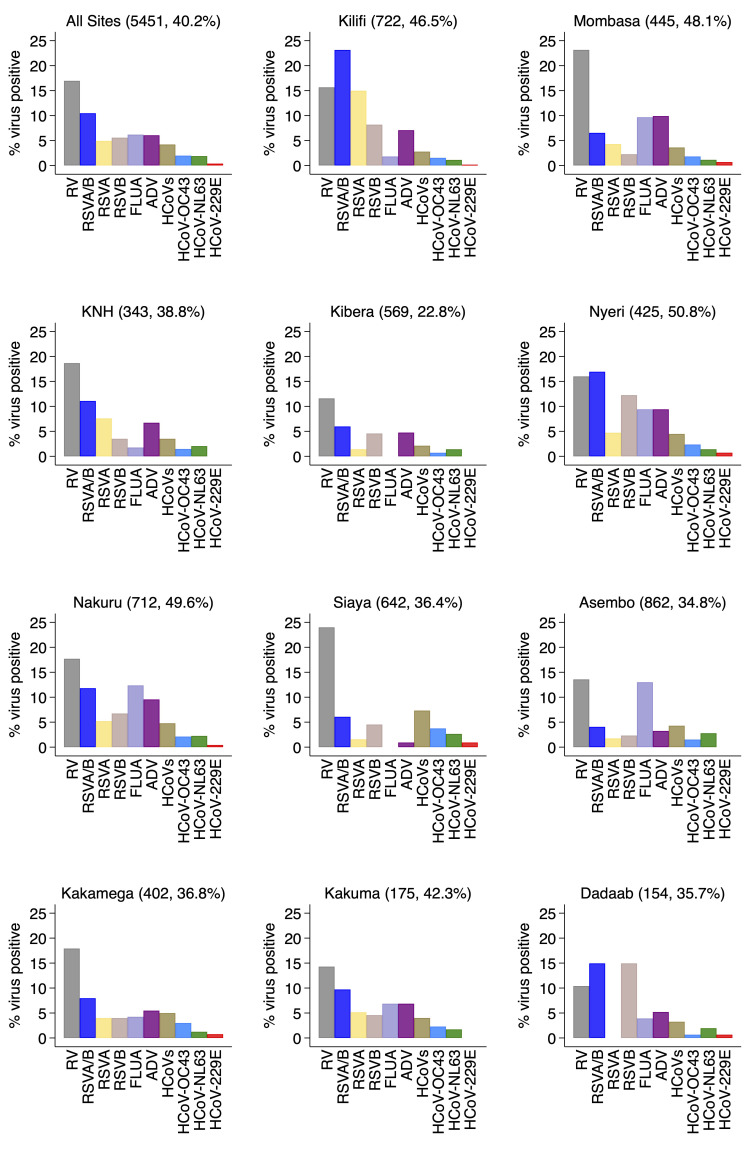
Percent virus positive for each of respiratory virus targets for the 11 surveillance sites (all sites together and each individually) in Kenya, January to December 2014. RSVA, RSV group A; RSVB, RSV group B; RSVAB, RSV group A or B; HRV, parainfluenza virus types 1–4; OC43, human coronavirus OC43; NL63, human coronavirus NL63; E229, human coronavirus E229; FLUA influenza virus types A; HCoV-OC43, human coronavirus OC43; HCoV-229E, human coronavirus 229E; HCoV-NL63, human coronavirus NL63; HCoVs, human coronavirus.

**Figure 4.**
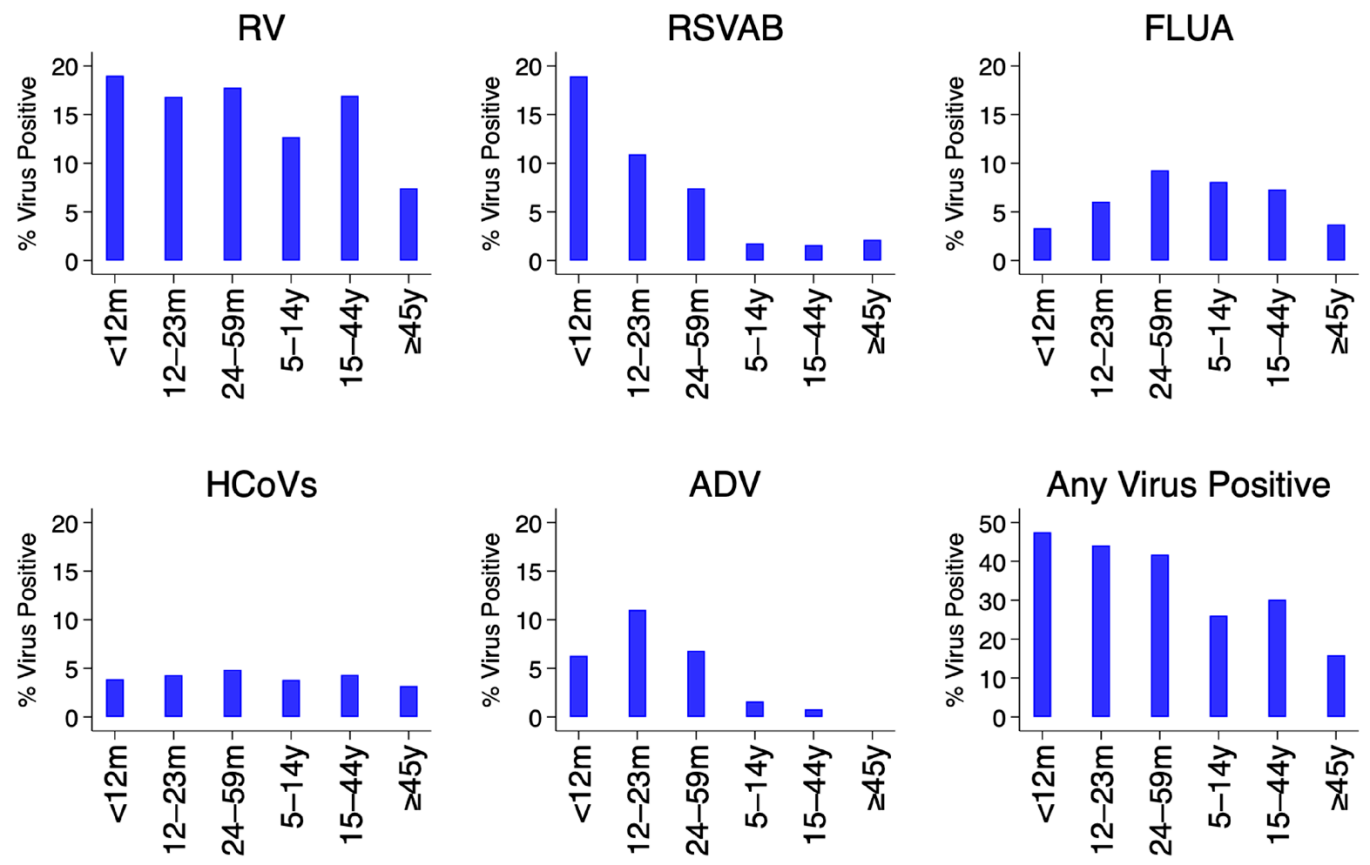
Percent of virus positives for each and all respiratory virus targets by age groups using the pooled data from the 11 surveillance sites in Kenya, January to December 2014. RSVA, RSV group A; RSVB, RSV group B; RV, rhinovirus; FLUA, influenza virus types A; HCoVs, human coronavirus; ADV, adenovirus.

For the individual virus targets, the most frequently detected were rhinoviruses (924, 17.0%) and RSV A/B (570, 10.5%). Other frequently detected viruses were human influenza virus A (337, 6.2%), Adenovirus (329, 6.0%) and hCoV (229, 4.2%) in that order ( Figure 3). The virus-specific percent positivity varied by surveillance site. For instance, the percent positive for RSV A/B was lowest in Asembo (4.1%) and highest in Kilifi (23.1%) while rhinoviruses were highest in Siaya (24.0%) and lowest in Dadaab (10.4%), Figure 3. The percent positivity of RSV and adenoviruses detections was age-dependent unlike rhinoviruses, hCoVs and influenza virus A. For RSV A/B, the highest positivity was among infants (18.9%) and lowest among older children (≥5 years) and adults (2.1%), while for adenoviruses highest positivity was among children aged 12–23 months (11.0%) and under one percent among participants aged ≥15 years old, Figure 4.

### Temporal patterns of detected viruses

**Figure 5.**
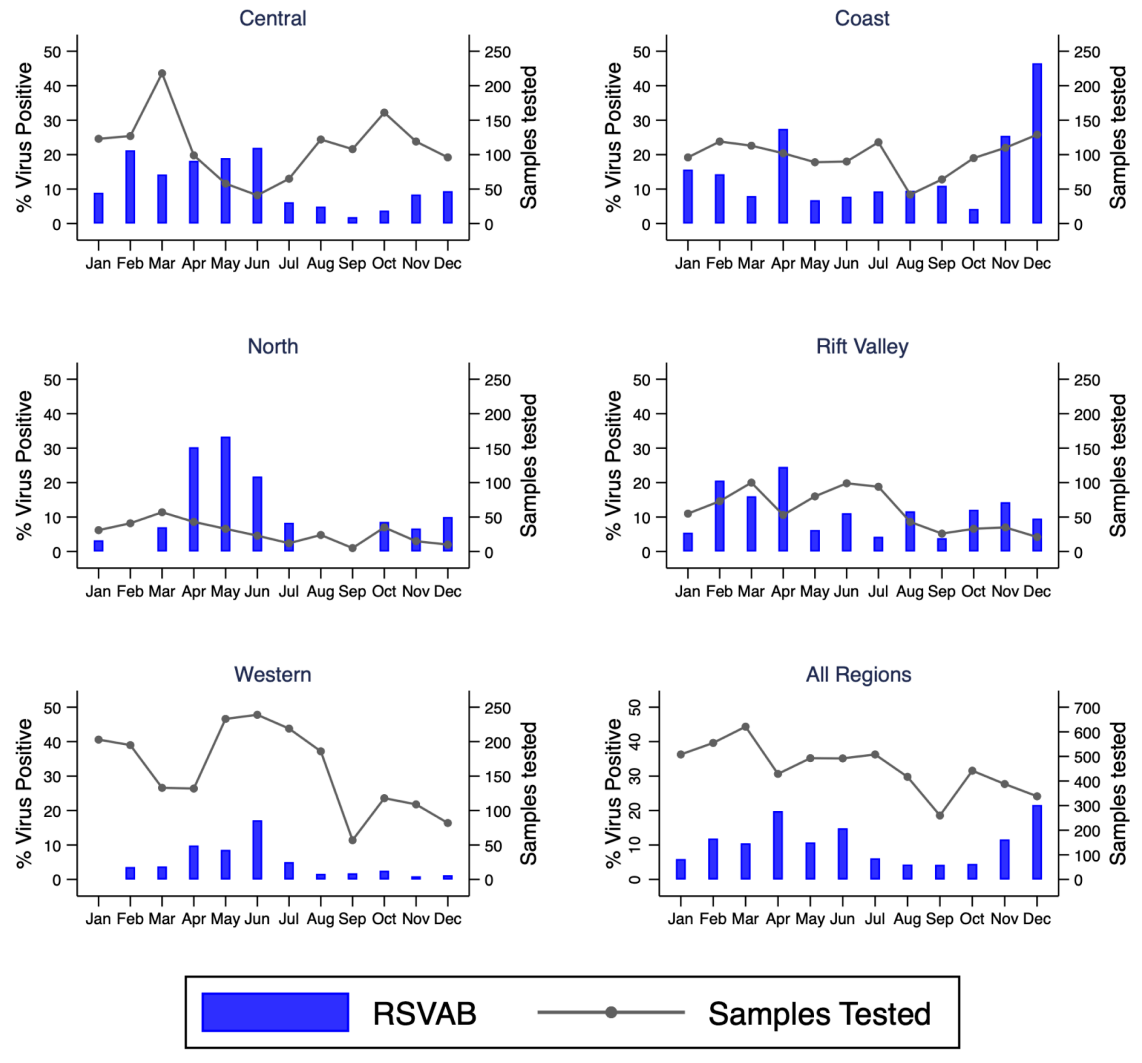
Percent of RSVAB virus positives by month for each region and overall in Kenya, January to December 2014. RSVAB, RSV group A or B; Sample Tested, samples tested.

We observed varying intensities in the circulation of the target respiratory viruses over the one year by surveillance sites or regions. Both RSV Group A and Group B co-circulated in all the sites except in Dadaab where only RSV group B was detected. RSV Group A detection predominated in Kenyatta National Hospital (KNH), Kilifi, and Mombasa while RSV B predominated in Kibera, Nyeri, Nakuru, and Siaya. For the rest of the sites the positivity of RSV A and B circulation was similar ( Figure 3). Monthly virus detections differed considerably by location for RSV A and B ( Figure 5 and *Extended data*: Supplementary File, Supplementary Figure 4 and 5) ^21^. Four peaks were observed for RSV A/B in Kenya: February (KNH, Kibera), April (Kilifi, Mombasa, Nyeri, Nakuru), May (Dadaab) and June (KNH, Kakamega, Siaya, Asembo, Mombasa, Kakuma). In November and December there was resurgence of RSV A/B circulation in some sites including Kilifi and Nyeri though the observation seemed to be truncated by the end of the study period.

Influenza virus A showed different circulation patterns by surveillance site. KNH, Asembo, Kakamega, and Dadaab had one peak in July, August, September, and November, respectively. Two peaks of influenza virus A were observed in Kakuma (May and July), Mombasa (May and September) and Nyeri (July and November). Adenoviruses and hCoVs didn’t show obvious variation in circulation over the study period (Extended data: Supplementary File, Supplementary Figure 4). However, there was a subtle increase in virus activity in some sites for hCoVs in July and August. Rhinoviruses circulated throughout the year with synchronous intensity across the country, including a reduced circulation in April. An exception was observed in Dadaab where the reduced rhinovirus activity was observed in July to September.

### Regional variation of virus circulation

All tested respiratory viruses were observed in all five regions (Rift valley, Coastal, North, Western, and Central). The highest and lowest detection of RSV A/B were observed in Coastal (16.8%) and Western (5.6%) region, respectively ( Figure 5, *Extended data*: Supplementary File, Supplementary Figure 6) ^21^. For influenza virus A, the highest positivity was observed in Rift Valley (11.3%) and the lowest in Central (0.7%) region ( *Extended data*: Supplementary File, Supplementary Figure 6) ^21^. Likewise, adenovirus had the highest detection in Rift Valley (9.5%) and lowest in the Western region (2.9%). Rhinovirus circulated with the highest prevalence across all regions with the Central (14.3%) and North (12.5%) region having slightly lower prevalence (Extended data: Supplementary File, Supplementary Figure 6) ^21^.

## Discussion

We report an overall virus positivity of 40.2% similar to an earlier report from Coastal Kenya (42.2% among person of all ages) ^22^ but lower than estimates from rural western Kenya (68% among person >5 years old) ^16^ and urban informal settlement in Nairobi (71.0% among children <5 years old) ^23^. Notably, there was considerable heterogeneity in the prevalence of virus detections (range, 22.8–50.8%) by surveillance site. Outpatient sites, Kibera and Asembo, had lower prevalence overall for RSV detections compared to inpatient settings. Detection of the target respiratory viruses was most common among young children. Monthly virus detection differed considerably by pathogen and geographic region. For instance different peaks of RSV A/B were observed in different regions at different months while rhinoviruses circulated throughout the year with synchronous intensity across the various regions. Taken together, these findings provide a better understanding of how the circulation of respiratory viruses varies by age, place, and over time. This underscores the need for subnational data on virus circulation to inform the design and prioritization of public health interventions including vaccines.

Human rhinoviruses (17.0%), RSV (10.5%) and influenza virus A (6.7%) viruses were the most detected respiratory viruses. Similar observations in other surveillance studies have been reported ^22, 24^. Even though the studies were carried out in different settings under different case definitions, they still show the three respiratory viruses as the most predominant respiratory viruses. In terms of their aetiological role, 31.1%, 2.0% and 7.5% of pneumonia were attributable to RSV, influenza and human rhinoviruses, respectively, in an international case-control study carried out in nine countries including Kenya ^6^. RSV has been strongly associated with hospitalized (severe) lower respiratory tract illnesses in other studies ^3– 6^ which concurs well with our observation of higher RSV positivity in inpatient settings compared to outpatient clinics. However, these studies are not directly comparable to our study because of differences in case definitions used and varying age groups included.

There was considerable variation of respiratory virus circulation across the country particularly for RSV and influenza virus. Human rhinovirus circulated throughout the year with strong synchrony across the geographic regions. Other respiratory viruses were more sporadic, with no obvious temporal pattern. The timing of RSV season differed between surveillance sites across Kenya but was similar in sites within the same region. The peak of RSV occurred in April in Coastal and Central Kenya sites, in May in Northern Kenya and in June in Western Kenya sites. These findings are consistent with previous studies in Kilifi ^17, 25^ and western Kenya ^14^ which reported data from multiple years ^25^. In Nairobi, we observed two peaks: one in February in Kibera and KNH and a second peak in June in KNH only. A similar surveillance at KNH in 1981 reported May–July as the peak months ^26^ while more recent analysis in the same hospital was unable to identify a defined RSV peak ^25^. Detections of RSV at KNH may not depict local circulation of the virus in Nairobi since the national referral hospital serves patients from all regions across the country. Our findings are consistent with those from a systematic analysis of the monthly virus activity worldwide by You Li *et al*., 2019 ^27^. Taken together the findings show that it is important to have a robust surveillance year-round in different parts of the country as there may be important sub-national variations in circulation patterns (as observed for RSV) that may need to be considered when deciding on trial sites and later when deciding on programmatic aspect of vaccine implementation especially for vaccine offering short duration of protection.

Specific respiratory viruses often co-circulate within the population. Of all detected respiratory viruses, 9.7% were co-detected. Of these, rhinovirus was most frequently identified with other viruses (6.7%) and influenza virus A least frequently (2.2%). The high frequency of rhinovirus co-detection is not surprising, as it was detected throughout the year with highest prevalence compared to other viruses. Though our study was not designed to examine the aetiologic contribution of codetections to respiratory disease, it is plausible these estimates of coinfection will be critical in interpreting future surveillance studies especially those conducted after implementation of targeted public health interventions such as vaccines. These data also inform various stakeholders, on whether to prioritize development and introduction of a vaccine that offers cross protection against multiple respiratory viral infections or to consider co-administration of the vaccines.

The prevalence of RSV and adenoviruses differed between age groups unlike human rhinoviruses, coronaviruses, and influenza virus A. For RSV, the highest prevalence was among infants and lowest among patients aged ≥5 years, while for adenoviruses highest prevalence was among children aged 12–23 months and under 1% among participants aged ≥15 years old. These results are consistent with observations from a respiratory surveillance study carried out in the outpatient setting in rural coastal Kenya among persons of all age groups ^22^. The study showed the highest-burden RSV and adenovirus to be within the younger age group making them the most vulnerable group for focus in future vaccine development and implementation. Low prevalence of respiratory viruses among adults might be due to low care-seeking behaviour, making them under-represented in the SARI surveillance sites, and by the time they get hospitalized, detection of a respiratory pathogen may be less likely. For other respiratory viruses, there was no differential prevalence by age.

Despite the study providing a countrywide perspective in respiratory virus circulation, we would like to point out a few limitations. First, we used specimens from only one calendar year and some participants were excluded from the analytical dataset, and year to year variations in respiratory virus circulation as well as variation in disease severity might have impacted the findings. A multi-year surveillance for these pathogens in geographically diverse regions would be warranted. Second, there were varying methods by sites, for instance, the disease case definition and ages enrolled. However, the varying methods would mainly affect the virus positivity estimates but not the temporal patterns observed over the one-year surveillance period. Thirdly, testing stored NP or/and OP could be inhibited by viral RNA. However, aliquots used in this study had at most been freeze-thawed once and tested within three years from date of collection. Fourth, data come from health facilities settings and do not necessarily reflect circulation of viruses in the community. Lastly, our findings can only infer on the most detected respiratory viruses but not the aetiological role that each virus plays.

## Conclusions

In conclusion, we identify human rhinoviruses, RSV, and influenza A as the most prevalent respiratory viruses among persons with acute respiratory illnesses in the one-year surveillance study. Long term surveillance is required to delineate the seasonal variations of respiratory viruses in multiple sites in a country over multiple years to inform design, development, and delivery strategies for optimal impact of public health interventions such as maternal vaccines and monoclonal therapy with short duration of action. Our data show that optimal implementation of virus-specific interventions such as vaccines may vary in different epidemiologic settings and subnational data are needed. Also, higher RSV positivity in inpatient settings compared to outpatient clinics strengthen the case for RSV vaccination in the future.

## Consent

Written informed consent for publication of the patients’ details was obtained from the patients or parents of the patient.

## Data Availability

Harvard Dataverse: Replication Data for: Surveillance of respiratory Viruses at health facilities from across Kenya, 2014. https://doi.org/10.7910/DVN/VFCZN4 ^21^. This project contains the following underlying data: Data file 1: 1_SPRED_Kenya_Descriptive_Analysis_07032021.do. Data file 2: 2_SPRED_Kenya_Viral_Trends_Site_07032021.do. Data file 3: 3_SPRED_Kenya_Viral_Trends_Region_07032021.do. Data file 4: NMurunga_SPRED_Kenya_descriptive_analysis_data_Codebook_V3.pdf. Data file 5: NMurunga_SRED_Kenya_descriptive_analysis_data_readme.txt. Data file 6: SRED_Kenya_descriptive_analysis_graph_generation_anon_v3-1.tab. Data file 7: SRED_Kenya_descriptive_analysis_graph_generation_anon_v3.tab. Harvard Dataverse: Replication Data for: Surveillance of respiratory Viruses at health facilities from across Kenya, 2014 https://doi.org/10.7910/DVN/VFCZN4 ^21^. This project contains the following extended data: Supplementary figures: Resp Virus Circulation in Kenya Supplementary Figures 07Apr2022.docx. Data are available under the terms of the Creative Commons Attribution 4.0 International license (CC-BY 4.0).
