# Supplementary material for: Surveillance of respiratory viruses at health facilities from across Kenya, 2014
Source: Wellcome Open Res. 2025 Dec 11;7:234. Originally published 2022 Sep 15. [Version 4] doi: 10.12688/wellcomeopenres.17908.4 (PMC12848351; doi:10.12688/wellcomeopenres.17908.4)
Supplement: Peer review history

# Reviewer report: Reviewer response for version 4

**DOI:** 10.21956/wellcomeopenres.28024.r144052

**Published:** 2026-01-27

**Authors:** Rino Rappuoli

**Affiliations:** 1 Fondazione Biotecnopolo di Siena, Siena, Italy

Respiratory infections are still the most common cause of mortality of children in low- and middle-income countries (LMICs). Given the implementation, in most of these countries, of wide vaccination programs against the most important causes of bacterial respiratory infections (e.g., *Haemophilus influenzae* type b and *Streptococcus pneumoniae*), the majority of these infections are now caused by viral agents. Accordingly, surveillance studies, at national and subnational level, are very important to better evaluate the epidemiology of viral infections in children, including their seasonality and patterns in the different ecosystems. Availability of these data will ultimately best inform public health interventions for the prevention of these infections and will provide stronger support for the use of well established, but still poorly used, respiratory vaccines and of recently introduced novel important preventive tools, like vaccines and monoclonal antibodies against the Respiratory Syncytial Virus (RSV).

 The paper “Surveillance of respiratory viruses at health facilities from across Kenya, 2014” by Nickson Murunga et al. describes a retrospective detection of various respiratory viruses from oropharyngeal and nasopharyngeal swabs obtained, in addition to demographic and clinical data, from subjects belonging to various age groups in 11 health facilities for either in-patients or out-patients and located in various regions of Kenya.

 As clearly indicated by the excellent reviews on the previous versions of the manuscript, this paper had some obvious limitations, partly due to the retrospective design of the study, which include: i) the duration of surveillance limited to just one calendar year, ii) the poorly standardized procedures for enrolment and iii) the differences in the data obtained from participating subjects in the various facilities. Despite these limitations, the study provides some important information. Firstly, the overall testing of the specimens showed that 40.2% were positive to at least one of the target viruses, that virus positive patients were significantly younger than negative patients and that the highest incidence of positivity was in infants <12 months. Secondly, virus detections were significantly higher in inpatient health facilities than in outpatient facilities, particularly for RSV, rhinovirus and adenovirus. Thirdly, the magnitude of prevalence by age was different for the tested viruses with RSV being the most prevalent in infants <12 months, adenovirus in children aged 12-23 months and influenza in children aged 24-59 months. Finally, some differences were observed among the 5 Kenyan regions evaluated in the study for overall prevalence of the various viruses and for seasonality. All this information provides useful insights for programmatic purposes.

 In conclusion, considering the valuable data produced and the diligence of the authors to either address the comments of the reviewers of the previous versions or to clearly describe in the manuscript the study limitations suggested by reviewers, I believe that this fourth version of the paper is mature for full endorsement, with the hope that readers of this manuscript from other LMIC are encouraged to implement surveillance studies for respiratory infections in their countries adopting a prospective design, well standardized procedures and data collection and possibly for a longer time that allows to detect a potential year to year variation in the circulation of the pathogens.

Is the work clearly and accurately presented and does it cite the current literature?

Yes

If applicable, is the statistical analysis and its interpretation appropriate?

Not applicable

Are all the source data underlying the results available to ensure full reproducibility?

Yes

Is the study design appropriate and is the work technically sound?

Yes

Are the conclusions drawn adequately supported by the results?

Yes

Are sufficient details of methods and analysis provided to allow replication by others?

Yes

Reviewer Expertise:

vaccinology

I confirm that I have read this submission and believe that I have an appropriate level of expertise to confirm that it is of an acceptable scientific standard.

# Reviewer report: Reviewer response for version 3

**DOI:** 10.21956/wellcomeopenres.22125.r66632

**Published:** 2023-11-02

**Authors:** W. Abdullah Brooks

**Affiliations:** 1 Department of International Health, Johns Hopkins Bloomberg School of Public Health, Baltimore, MD, USA

I have reviewed the revised (version 3) of the above - referenced manuscript. The authors highlight several of the changes made since version 2. Descriptively, these appear to be responsive to earlier reviewers’ comments/suggestions. 

 I support the indexing of this paper with no further suggestions for revision.

Is the work clearly and accurately presented and does it cite the current literature?

Yes

If applicable, is the statistical analysis and its interpretation appropriate?

No

Are all the source data underlying the results available to ensure full reproducibility?

Yes

Is the study design appropriate and is the work technically sound?

No

Are the conclusions drawn adequately supported by the results?

Yes

Are sufficient details of methods and analysis provided to allow replication by others?

Yes

Reviewer Expertise:

NA

I confirm that I have read this submission and believe that I have an appropriate level of expertise to confirm that it is of an acceptable scientific standard.

# Reviewer report: Reviewer response for version 3

**DOI:** 10.21956/wellcomeopenres.22125.r66631

**Published:** 2023-10-23

**Authors:** David P Moore

**Affiliations:** 1 Vaccine and Infectious Diseases Analytics (VIDA) Research Unit, University of the Witwatersrand, Johannesburg, South Africa; 2 Department of Paediatrics and Child Health, Chris Hani Baragwanath Academic Hospital, Faculty of Health Sciences, University of the Witwatersrand, Johannesburg, South Africa

Thank you for the opportunity of reviewing the revised version of this manuscript. The authors highlight patterns of respiratory virus detection across 11 sites in Kenya during the 2014 calendar year. While considerable regional differences in virus detection occurred, a consistent finding was that of respiratory syncytial virus (RSV) detection in the youngest age groups, and in hospitalised patients, and adenovirus detection in children 12-23 months of age. The authors motivate for establishment of a national surveillance platform to track trends in respiratory virus isolation using standardised case definitions. Such a platform would be important in the lead up to introduction of candidate vaccines to prevent respiratory viral hospitalisations.

 Major comments:

1. The fact that RSV was more prevalent in hospitalised participants is not highlighted in the Results and Discussion sections. It would be useful to include a Table with viral detection prevalence stratified by inpatient versus outpatient status.
2. Death as an outcome is not described in this study. How many of the participants had an outcome of death? It would be useful to include a Table with viral detection prevalence stratified by survival status.
3. How did viral co-infection impact on hospitalisation and outcome status? It would be useful to include this in the suggested tables mentioned above.

Minor comments:

1. In the Abstract, mention in the results section that RSV was more prevalent in inpatients compared to outpatients.
2. In the first paragraph of the Introduction, suggest omit the word “recent” in the sentence which highlights the introduction of bacterial conjugate vaccines.

Is the work clearly and accurately presented and does it cite the current literature?

Yes

If applicable, is the statistical analysis and its interpretation appropriate?

Yes

Are all the source data underlying the results available to ensure full reproducibility?

Yes

Is the study design appropriate and is the work technically sound?

Yes

Are the conclusions drawn adequately supported by the results?

Partly

Are sufficient details of methods and analysis provided to allow replication by others?

Yes

Reviewer Expertise:

Paediatric infectious diseases; paediatric pneumonia; antimicrobial stewardship

I confirm that I have read this submission and believe that I have an appropriate level of expertise to confirm that it is of an acceptable scientific standard, however I have significant reservations, as outlined above.

# Response

**Published:** 2025-12-09

**Authors:** Nickson Murunga

**Affiliations:** Kenya Medical Research Institute (KEMRI)-Wellcome Trust Research Programme, Kenya

Major comments: The fact that RSV was more prevalent in hospitalised participants is not highlighted in the Results and Discussion sections. It would be useful to include a Table with viral detection prevalence stratified by inpatient versus outpatient status.

- **Response: **We thank the reviewer for this observation. We have updated the Results section to include Table 5, which presents viral detection prevalence stratified by inpatient and outpatient setting, and revised the accompanying text to highlight the higher RSV prevalence among hospitalised participants. This table, together with our earlier supplementary material (Extended data: Supplementary File, Supplementary Figure 1), provides summary of prevalence patterns across the two settings.

Death as an outcome is not described in this study. How many of the participants had an outcome of death? It would be useful to include a Table with viral detection prevalence stratified by survival status.

- **Response: **We appreciate the reviewer’s comment and agree that such a summary would have been valuable for understanding disease severity. However, due to the retrospective nature of our testing and the lack of standardised data collection across sites, outcome information was incomplete across sites and insufficient for comparing viral detection by survival status. This limitation is acknowledged in the Discussion.

How did viral co-infection impact on hospitalisation and outcome status? It would be useful to include this in the suggested tables mentioned above.

- **Response:** *We have now included Table 5, which presents viral co-detection patterns stratified by inpatient and outpatient facilities. However, as noted in the Discussion, outcome data were incomplete across surveillance sites and could not be reliably incorporated into this summary.*

Minor comments: In the Abstract, mention in the results section that RSV was more prevalent in inpatients compared to outpatients.

- **Response:** *Thank you for the suggestion. We have now included this information in the abstract.*

In the first paragraph of the Introduction, suggest omit the word “recent” in the sentence which highlights the introduction of bacterial conjugate vaccines.

- **Response:** *Thank you for the suggestion. The word “recent” has now been omitted from the Introduction as recommended.*

# Reviewer report: Reviewer response for version 2

**DOI:** 10.21956/wellcomeopenres.20410.r53046

**Published:** 2022-11-23

**Authors:** W. Abdullah Brooks

**Affiliations:** 1 Department of International Health, Johns Hopkins Bloomberg School of Public Health, Baltimore, MD, USA

Summary:

 The authors point out what has emerged as the consensus view that viruses have assumed greater prominence in ARI aetiology with the increased uptake of vaccines against bacterial ARI agents. This, in turn, requires global geographically representative data on virus circulation.

 They used three health facility-based respiratory illness surveillance platforms across 11 sites in Kenya, collecting NP and OP swabs, patient-level demographic and clinical data obtained in 2014 at various surveillance facilities. RT-PCR was used to identify RV, RSV, influenza, hCoV and adenoviruses.

 They report the results from 11 sites yielding 5451 NP/OP specimens. 40.2% were positive for at least one of the target viruses. The most frequently detected included HRV (17.0%), RSV A/B (10.5%), influenza A (6.2%) and hCoV (4.2%). RSV was most prevalent in infants <12m, adenovirus in children 12-23m, and HRV across all age groups. They report that the overall percent of virus positivity varied by surveillance site, health facility type and case definition used in the surveillance system.

 They conclude that HRV, RSV, and influenza A are the most prevalent respiratory viruses in their populations, and that higher RSV positivity in inpatient settings strengthens the case for RSV vaccination, and that longterm surveillance is necessary to establish regional heterogeneity in respiratory virus circulation and seasonality. 

 General comments:

 The paper is well-written and referenced with appropriate and representative citations. Many of the authors are well-published in the field, with several of the involved sites contributing substantially to the respiratory disease literature over time. There are, however, a few outstanding questions from the manuscript.

 In the methods section, regarding patient enrolment, given the heterogeneity of enrolment criteria between sites, did the investigators conduct any exercise to determine whether or not, and what fraction, of patients enrolled in one setting might be omitted from another, or whether there could be substantial systematic syndromic differences between patient populations? Although each of these 11 sites had substantial experience, other multi-centre studies, notably PERCH which the authors reference, used standardised enrolment criteria across all sites. Given the substantial differences in the prevalence of specific viruses by site, the question is to what extent did enrolment criteria affect virus selection?

 Staying with enrolment, was this conducted by project-trained staff or was enrolment per routine? If the latter, was any exercise conducted to determine what fraction of potentially eligible patients might have been missed by site? Second, regarding data collection, were demographic and clinical data collection forms harmonised between sites, or was this left to the individual sites? Given that the US CDC supported the influenza testing, why was influenza B not tested or at least not reported, as influenza B viruses have been shown to contribute substantially to childhood infection?

 Regarding mixed virus identification, how was infection assigned, or were all counted?

 Regarding virus circulation, was there any sampling by any site of non-respiratory patients in order to assess whether or not these viruses were also present in non-respiratory presentations?

 It is not uncommon for RSV to circulate differently by location, particularly when those locations are distinct [Stockman et al 2013]. As for influenza, it may commonly start in one region and then spread to others [Zaman, R. 2009]. Given the short duration of the study period, this directionality may not have been observed but was it looked for? Two influenza peaks are also not uncommon, and may have reflected distinct H1N1 and H3N2 patterns; was this looked for? Did the age groups suggest how to adjust surveillance in order to provide a representative assessment of disease burden across sites and age groups, and if so, in what manner? For example, if certain sites used criteria that selected against older or younger age groups, was consideration given to expanding those criteria according to experiences in other sites?

 The other study limitations have been well-described by the authors.

 Specific comments:

 None.

Is the work clearly and accurately presented and does it cite the current literature?

Yes

If applicable, is the statistical analysis and its interpretation appropriate?

No

Are all the source data underlying the results available to ensure full reproducibility?

Yes

Is the study design appropriate and is the work technically sound?

No

Are the conclusions drawn adequately supported by the results?

Yes

Are sufficient details of methods and analysis provided to allow replication by others?

Yes

Reviewer Expertise:

NA

I confirm that I have read this submission and believe that I have an appropriate level of expertise to confirm that it is of an acceptable scientific standard, however I have significant reservations, as outlined above.

# Response

**Published:** 2023-08-30

**Authors:** Nickson Murunga

**Affiliations:** Kenya Medical Research Institute (KEMRI)-Wellcome Trust Research Programme, Kenya

General comments:

 The paper is well-written and referenced with appropriate and representative citations. Many of the authors are well-published in the field, with several of the involved sites contributing substantially to the respiratory disease literature over time. There are, however, a few outstanding questions from the manuscript.

 In the methods section, regarding patient enrolment, given the heterogeneity of enrolment criteria between sites, did the investigators conduct any exercise to determine whether or not, and what fraction, of patients enrolled in one setting might be omitted from another, or whether there could be substantial systematic syndromic differences between patient populations? Although each of these 11 sites had substantial experience, other multi-centre studies, notably PERCH which the authors reference, used standardised enrolment criteria across all sites. Given the substantial differences in the prevalence of specific viruses by site, the question is to what extent did enrolment criteria affect virus selection?

*Thank you for emphasizing this critical aspect. Given the retrospective nature of our study involving samples from different sites, there was no pre-established standardization of clinical case definitions. We recognize that the varying methodologies among sites could influence virus positivity estimates, although not greatly affecting the temporal patterns observed during the one-year surveillance period. The need for a standardized data collection platform to provide robust accurate representation of virus positivity across sites is apparent and we now highlight this in the discussion. However, the current data still offers valuable insights into potential temporal patterns and how they vary geographically in Kenya albeit for one year.*

 Staying with enrolment, was this conducted by project-trained staff or was enrolment per routine?

*Project-trained surveillance officers conducted the study , and individuals meeting the inclusion criteria in each site were approached for consenting and enrolment.*

 If the latter, was any exercise conducted to determine what fraction of potentially eligible patients might have been missed by site? Second, regarding data collection, were demographic and clinical data collection forms harmonised between sites, or was this left to the individual sites?

*Thank you for raising this crucial point. We have acknowledge this limitation within our study. Given the retrospective nature of our analysis, the data collection process was not standardized in advance and in some sites we do not have the full dataset with all eligible participants. However, the dedicated surveillance officers ensured identification of eligible patients, and upon consenting collected the NP/OP. We do not envisage substantial variations in enrollment occurred across the sites.*

 Given that the US CDC supported the influenza testing, why was influenza B not tested or at least not reported, as influenza B viruses have been shown to contribute substantially to childhood infection?

*Thank you for bringing up this matter. We conducted testing for influenza B across all the sites; however, it's important to note that during the study period, there was a scarcity of identified influenza B viruses across these sites. Throughout the entire duration, a mere 36 cases of influenza B positivity were detected from  the 11 sites. We found this number to be exceptionally low for meaningful comparison.*

*Regarding mixed vi*rus identification, how was infection assigned, or were all counted?

*Thank you for raising this concern. In terms of identifying mixed viruses, the approach taken was to consider co-detection as a sample tested positive for two or more respiratory viruses. We counted all instances of co-detection for various combinations. For example, if one sample was tested for both RSV Group A and rhinovirus and another sample from a different individual exhibited the same co-detection, this scenario was recorded as two instances of RSVA and Rhino detection. Every positive result for the targeted viruses was treated as a potential infection for the respective participants.* *The information raised has been addressed by making amendments to the methodology section of the manuscript. Furthermore, we have included a supplementary figure in the results section to provide additional context.*

 Regarding virus circulation, was there any sampling by any site of non-respiratory patients in order to assess whether or not these viruses were also present in non-respiratory presentations?

*Non-respiratory patients were not part of our sampling process, as they were not part of the original survey's objectives. It would have been great for assessing aetiologic role of the detected respiratory viruses.*

 It is not uncommon for RSV to circulate differently by location, particularly when those locations are distinct [Stockman et al 2013]. As for influenza, it may commonly start in one region and then spread to others [Zaman, R. 2009]. Given the short duration of the study period, this directionality may not have been observed but was it looked for?

*Thank you for highlighting this important point. We did not analyze the directionality of transmissions in our study. The data collection sites are spread out and cover a wide geographic area. For example, sites in Nairobi and Nakuru are not adjacent to each other. This geographical distribution makes it difficult to outline clear transmission pathways based solely on virus positivity, such as the initiation of influenza in Nakuru and its subsequent spread. With only 11 sites, which might not be fully representative of the entire country, it is likely that we missed out on capturing comprehensive transmission pathways. Additionally, our study spanned only one year, making it difficult to observe intricate patterns. A longer study duration would be necessary to better capture such complex transmission dynamics and pathways. * Two influenza peaks are also not uncommon, and may have reflected distinct H1N1 and H3N2 patterns; was this looked for? *We also didn’t perform part of whole genome sequencing which would also help delineate possible transmission pathways as you note for H1N1 and H3N2 patterns.*

 Did the age groups suggest how to adjust surveillance in order to provide a representative assessment of disease burden across sites and age groups, and if so, in what manner? For example, if certain sites used criteria that selected against older or younger age groups, was consideration given to expanding those criteria according to experiences in other sites?

*We did not adjust for the age differences of the enrolled participants by site. We aimed to focus more on temporal patterns and their differences across sites in this manuscript rather than compare disease burden.  An alternate study design would be required for accurate estimation of disease burden.*

 The other study limitations have been well-described by the authors.

# Reviewer report: Reviewer response for version 1

**DOI:** 10.21956/wellcomeopenres.19845.r52434

**Published:** 2022-09-30

**Authors:** David P Moore

**Affiliations:** 1 Vaccine and Infectious Diseases Analytics (VIDA) Research Unit, University of the Witwatersrand, Johannesburg, South Africa; 2 Department of Paediatrics and Child Health, Chris Hani Baragwanath Academic Hospital, Faculty of Health Sciences, University of the Witwatersrand, Johannesburg, South Africa

Thank you for the opportunity of reviewing this excellent paper by a well-established research group, tracking the burden and geographic variability of respiratory viral carriage across 11 sites in Kenya.

**Major comments:**

1. Collection over one calendar year is limiting considering the seasonal variation in carriage and infection rates related to circulating respiratory viruses. Temporal and regional patterns of respiratory virus activity cannot be adequately appreciated in a 12 month analysis. This limitation has been highlighted by the authors, and should serve as a strong motivation for ongoing, multi-year surveillance of virus activity across Kenya.
2. What justification was applied in applying test positivity only to PCR reactions that had Ct values of < 35? This should be referenced in the Methods section.
3. Relatively sparse representation of sites from the northeast of Kenya is a concern, and should be addressed in future respiratory virus surveillance activities by this research group.
4. Mention is made of informed consent. Was assent applied to children old enough to give assent? If so, this should be mentioned in the manuscript.
5. Temporal shifts in RSV serotype predominance cannot be adequately interpreted from the data presented in this manuscript, as a large proportion of RSV detections were of the combined “RSVA/B” category. If data are available to appreciate the RSV serotype in all tested specimens, then it would be appropriate to present a dichotomised “RSVA” and “RSVB” analysis; however, if these data are not available for all sites, I would recommend that the authors merely present a consolidated “RSV” summary prevalence for each site.
6. A formal analysis of the test positivity rates for RSV in inpatient compared to outpatient settings is not presented in the Results section, yet is prominently mentioned in the Abstract, Discussion and Conclusion sections of the manuscript. It is important to present this analysis in results, in order to justify the authors’ assertion that RSV vaccine delivery would be expected to impact favourably on hospitalisation rates, once such a vaccine becomes available.

**Minor comments:**

1. Abstract: In the Conclusions, mention is made of the fact that respiratory syncytial virus (RSV) was more prevalent in inpatients. This finding is not presented in the Abstract Results, and should be presented there. Additionally, consider refining the sentence in the Conclusions to indicate that RSV was more prevalent in infants. Consider using: “Higher RSV positivity in inpatients, and in infants, strengthens the case for RSV vaccination.”
2. Presentation of RSVA/B, RSVA and RSVB bars in Figure 3 muddies the interpretation of RSV prevalence at each site somewhat. Suggest collapse these into one “RSV” bar for presentation in the main paper, and move the serotype specific RSV bar charts to Supplementary Materials.
3. Presentation of HCoVs, HCoV-OC43, HCoV-NL63 and HCoV-229E bars in Figure 3 muddies the interpretation of HCoV prevalence at each site somewhat. Suggest collapse these into one “HCoV” bar for presentation in the main paper, and move the serotype specific HCoV bar charts to Supplementary Materials.
4. Include “ADV, adenovirus” and “RV, human rhinovirus” in the legend to Figure 3.
5. Remove “HRV, parainfluenza virus types 1-4; OC43, human coronavirus OC43; NL63, human coronavirus NL63; E229, human coronavirus E229” from the legend in Figure 3.
6. Remove “RSVA, RSV group A; RSVB, RSV group B” from the legend in Figure 4, and replace with “RSV”.
7. Results section, second last paragraph: replace “didn’t” with “did not”.
8. In the Discussion, second paragraph on page 13, suggest reword to “Taken together the findings show that it is important to have robust, ongoing, year-round surveillance in different parts of the country as there may be important sub-national variations in circulation patterns…”

Is the work clearly and accurately presented and does it cite the current literature?

Yes

If applicable, is the statistical analysis and its interpretation appropriate?

Yes

Are all the source data underlying the results available to ensure full reproducibility?

Yes

Is the study design appropriate and is the work technically sound?

Yes

Are the conclusions drawn adequately supported by the results?

Partly

Are sufficient details of methods and analysis provided to allow replication by others?

Yes

Reviewer Expertise:

Paediatric pneumonia epidemiology and aetiology.

I confirm that I have read this submission and believe that I have an appropriate level of expertise to confirm that it is of an acceptable scientific standard, however I have significant reservations, as outlined above.

# Response

**Published:** 2023-08-30

**Authors:** Nickson Murunga

**Affiliations:** Kenya Medical Research Institute (KEMRI)-Wellcome Trust Research Programme, Kenya

1. Abstract: In the Conclusions, mention is made of the fact that respiratory syncytial virus (RSV) was more prevalent in inpatients. This finding is not presented in the Abstract Results, and should be presented there. Additionally, consider refining the sentence in the Conclusions to indicate that RSV was more prevalent in infants. Consider using: “Higher RSV positivity in inpatients, and in infants, strengthens the case for RSV vaccination.”

*Thank you for your valuable feedback. We have now updated the Abstract Results to include the finding that RSV was more prevalent in inpatients. Additionally, in the Conclusion section, we have emphasized the higher RSV positivity in inpatients and infants, which further strengthens the case for RSV vaccination.*

1. Presentation of RSVA/B, RSVA and RSVB bars in Figure 3 muddies the interpretation of RSV prevalence at each site somewhat. Suggest collapse these into one “RSV” bar for presentation in the main paper, and move the serotype specific RSV bar charts to Supplementary Materials.

*Thank you for your feedback. We have collapsed the RSVA/B, RSVA, and RSVB bars into one combined "RSV" bar for presentation in the main paper, as suggested. The serotype-specific RSV bar charts have been moved to the Supplementary Materials.*

1. Presentation of HCoVs, HCoV-OC43, HCoV-NL63 and HCoV-229E bars in Figure 3 muddies the interpretation of HCoV prevalence at each site somewhat. Suggest collapse these into one “HCoV” bar for presentation in the main paper, and move the serotype specific HCoV bar charts to Supplementary Materials.

*Thank you for your feedback. We have collapsed the HCoV-OC43, HCoV-NL63 and HCoV-229E bars into one combined " HCoV " bar for presentation in the main paper, as suggested. The serotype-specific HCoV bar charts have been moved to the Supplementary Materials.*

1. include “ADV, adenovirus” and “RV, human rhinovirus” in the legend to Figure 3.

*Thank you for your suggestion. We have now included "ADV, adenovirus" and "RV, human rhinovirus" in the legend to Figure 3.*

1. Remove “HRV, parainfluenza virus types 1-4; OC43, human coronavirus OC43; NL63, human coronavirus NL63; E229, human coronavirus E229” from the legend in Figure 3.

*Thank you for your suggestion. We have now removed “HRV, parainfluenza virus types 1-4; OC43, human coronavirus OC43; NL63, human coronavirus NL63; E229, human coronavirus E229” from the legend in Figure 3.*

1. Remove “RSVA, RSV group A; RSVB, RSV group B” from the legend in Figure 4, and replace with “RSV”.

*Thank you for bringing this to our attention. We have made the necessary corrections.*

1. Results section, second last paragraph: replace “didn’t” with “did not”.

*The suggested change has been effected. Thank you.*

1. In the Discussion, second paragraph on page 13, suggest reword to “Taken together the findings show that it is important to have robust, ongoing, year-round surveillance in different parts of the country as there may be important sub-national variations in circulation patterns…”

Revised accordingly.
